# An ensemble learning approach for modeling the systems biology of drug-induced injury

**DOI:** 10.1186/s13062-020-00288-x

**Published:** 2021-01-12

**Authors:** Joaquim Aguirre-Plans, Janet Piñero, Terezinha Souza, Giulia Callegaro, Steven J. Kunnen, Ferran Sanz, Narcis Fernandez-Fuentes, Laura I. Furlong, Emre Guney, Baldo Oliva

**Affiliations:** 1 Research Programme on Biomedical Informatics (GRIB), Hospital del Mar Medical Research Institute (IMIM), DCEXS, Pompeu Fabra University (UPF), Barcelona, Spain; 2 Department of Toxicogenomics, Maastricht University, Maastricht, The Netherlands; 3 Leiden Academic Centre for Drug Research, Leiden University, Leiden, The Netherlands; 4 Department of Biosciences, U Science Tech, Universitat de Vic-Universitat Central de Catalunya, Vic, Spain; 5 Institute of Biological, Environmental and Rural Sciences, Aberystwyth University, Aberystwyth, UK

**Keywords:** CAMDA, Drug-induced liver injury, Hepatotoxicity, Drug safety, Systems biology, Machine learning, Cmap, Drug structure

## Abstract

**Background:**

Drug-induced liver injury (DILI) is an adverse reaction caused by the intake of drugs of common use that produces liver damage. The impact of DILI is estimated to affect around 20 in 100,000 inhabitants worldwide each year. Despite being one of the main causes of liver failure, the pathophysiology and mechanisms of DILI are poorly understood. In the present study, we developed an ensemble learning approach based on different features (CMap gene expression, chemical structures, drug targets) to predict drugs that might cause DILI and gain a better understanding of the mechanisms linked to the adverse reaction.

**Results:**

We searched for gene signatures in CMap gene expression data by using two approaches: phenotype-gene associations data from DisGeNET, and a non-parametric test comparing gene expression of DILI-Concern and No-DILI-Concern drugs (as per DILIrank definitions). The average accuracy of the classifiers in both approaches was 69%. We used chemical structures as features, obtaining an accuracy of 65%. The combination of both types of features produced an accuracy around 63%, but improved the independent hold-out test up to 67%. The use of drug-target associations as feature obtained the best accuracy (70%) in the independent hold-out test.

**Conclusions:**

When using CMap gene expression data, searching for a specific gene signature among the landmark genes improves the quality of the classifiers, but it is still limited by the intrinsic noise of the dataset. When using chemical structures as a feature, the structural diversity of the known DILI-causing drugs hampers the prediction, which is a similar problem as for the use of gene expression information. The combination of both features did not improve the quality of the classifiers but increased the robustness as shown on independent hold-out tests. The use of drug-target associations as feature improved the prediction, specially the specificity, and the results were comparable to previous research studies.

**Supplementary Information:**

The online version contains supplementary material available at 10.1186/s13062-020-00288-x.

## Background

Drug safety is one of the main reasons of drug attrition during development [1, 2]. Although the causes of drug failure due to lack of safety are several, hepatic adverse reactions are among the most important, particularly at late drug development stages [3, 4]. Drug-induced liver injury (also named DILI) is an adverse reaction caused by the intake of drugs of common use that produces liver damage. DILI has a relatively high incidence rate, estimated to affect around 20 in 100,000 inhabitants worldwide each year [5]. Many drugs ranging from pain killers to anti-tuberculous treatments can cause DILI [6]. Despite DILI being one of the leading causes of acute liver failure, the pathophysiology and etiology of DILI is poorly understood and pinpointing the toxicity of compounds in human liver remains a non-trivial task [7].

Several *in-silico* methods have been proposed to predict hepatotoxicity of drugs. Among these, machine learning models trained using drug structural features have shown a good accuracy [8–10]. Furthermore, incorporating gene- and pathway-level signatures from transcriptomics data has shown a high predictive accuracy using Deep Neural Networks [11]. With the recent increased interest on machine learning methods to predict drug-induced toxicity, the International Conference on Critical Assessment of Massive Data Analysis (CAMDA) has been organizing the Connectivity Map (CMap) Drug Safety Challenge since 2018. The aim of the challenge was to assess the state-of-the-art on DILI prediction methods using different sources of data such as transcriptomics data, chemical structures, and cellular images. In the first edition (CAMDA 2018), the two published studies applied various machine learning methods for DILI prediction on the CMap gene expression data provided (in MCF7 and PH3 cell lines), obtaining poor predictive results [12, 13]. *Sumsion* et al. [12] evaluated 7 different classification algorithms and built a soft-voting classifier that combined all classifiers. Still, the accuracy results of the best performing classifiers (random forest and soft-voting) were around 70%, obtaining high sensitivity (77%) but low specificity (13–19%). They also explored different strategies to improve the results, such as normalizing gene expression data across samples, feature selection methods, adjusting class imbalance or improving the voting-based classifier. Still, the improvement of the results with each of these solutions was limited. *Chierici* et al. [13] used three deep learning classifiers and compared them with random forest and multi-layer perceptron classifiers. They also tested several strategies for balancing data and alternative train/test splits. However, the different strategies gave an overall poor performance, in which the Matthews correlation coefficient (MCC) values ranged from − 0.04 to 0.21 in cross-validation and − 0.16 to 0.11 in the independent hold-out test set. In both *Sumsion* et al. [12] and *Chierici* et al. [13], the limited results were attributed to having a small and highly imbalanced gold standard of 190 drugs for training (160 DILI-causing) and 86 drugs for an independent hold-out test. This problem is still present in the current edition of CAMDA (2019), as the size of the gold standard is still limited. The organizers provided a gold standard (from DILIrank dataset [14]) composed of 175 drugs for training and 55 for an independent hold-out test. They also provided a dataset of CMap L1000 gene expression responses for 1314 compounds [15] (including the 230 drugs of the gold standard), the chemical structures (SMILES codes) of the drugs and annotated images from cell perturbation assays for a subset of 826 compounds (156 from DILIrank) [16].

In this study, we implemented an ensemble learning approach to predict drugs that can cause DILI in human liver. We experimented the inclusion in the classifiers of several features derived from transcriptomics, drug-target associations and structural data either separately or combined (Table 1). We investigated whether it was feasible to find a DILI gene signature using phenotype-gene associations, protein-protein interactions and gene expression data. We observed that finding a meaningful gene signature can improve the quality of the classifier instead of using all landmark genes defined in the CMap platform (i.e. the subset of 978 genes whose gene expression has been determined as informative enough to characterize the whole transcriptome [15]). We also analyzed the accuracy of the prediction when using chemical structures, drug-target information, and the combination of these together with transcriptomics data. We compared the quality of the classifiers made from these features in a robust machine learning pipeline and presented a list of conclusions that might serve as starting points for further studies.

**Table 1:** Summary of the features used in the classification task

Type of feature	Name	Description
Gene expression features	Landmark genes	978 genes directly measured from the L1000 datasets
	DisGeNET DILI genes	Curated genes associated to 9 phenotypes related with DILI from DisGeNET database
	GUILDify DILI genes	Genes associated through the protein interactions network to 6 phenotypes related with DILI using GUILDify
	DILI landmark genes	66 landmark genes selected using non-parametric test for each gene across all samples of Most/Less- vs. No-DILI-Concern drugs (*P*-value< 0.05)
Structural features	SMILES	Line notation describing the chemical structure of drugs
Drug target genes	Set of targets	1664 drug targets retrieved from DGIdb, HitPick and SEA

## Methods

### Gold standard data on drugs causing DILI

The CAMDA challenge provided the DILIrank dataset [14] as the gold standard data of known DILI compounds. DILIrank is a dataset that classifies the drugs in three levels of DILI severity: “Most-DILI-Concern” when the drug was withdrawn for DILI-related causes or labelled with severe DILI indication; “Less-DILI-Concern” when the drug was labelled with mild DILI indication or adverse reactions; and “No-DILI-Concern” when no DILI was indicated in any of the labelling sections. Moreover, these levels of severity were verified using the standardized clinical causality assessment system, and the drugs that were not meeting the expected severity were reclassified as “Ambiguous-DILI-Concern”. Among all the drugs categorized in DILIrank, the CAMDA challenge provided data for 230 drugs: 37 Most-DILI-Concern, 87 Less-DILI-Concern, 51 No-DILI-Concern and 55 Ambiguous-DILI-Concern. Additionally, the US Food and Drug Administration classified the remaining 55 Ambiguous-DILI-Concern drugs as DILI or No-DILI-Concern. These 55 drugs served as a dataset for an independent hold-out test, because the actual severity category of the drug remained hidden.

### Data collection

#### CMap gene expression

The gene expression data used in this study was gathered from the CMap L1000 Assay Platform [15]. The L1000 Assay Platform provides more than one million gene expression profiles from a wide range of cell lines treated with different drugs at different doses and treatment durations. Assuming that gene expression is highly correlated, the Platform features a subset of approximately 1000 landmark genes to derive profiles that serve to infer the expression of the rest of genes. We used CMAP L1000 level 5 data which contained z-score values corresponding to the normalized differential expression between the drug treatment and control across different conditions.

#### Genes associated to DILI related phenotypes

We manually curated a list of phenotypes closely related with DILI and identified the genes associated with these phenotypes using the DisGeNET database v6.0 [17] (Table 2). We restricted disease-gene associations solely to expertly curated repositories: UniProt [18], the Comparative Toxicogenomics Database (CTD) [19], ORPHANET [20], the Clinical Genome Resource (CLINGEN) [21], the Genomics England PanelApp [22] and the Cancer Genome Interpreter (CGI) [23]. We kept only the phenotypes with at least 10 curated gene associations. The full list of associations between DILI phenotypes and genes can be found at Supplementary Table 1.

**Table 2:**
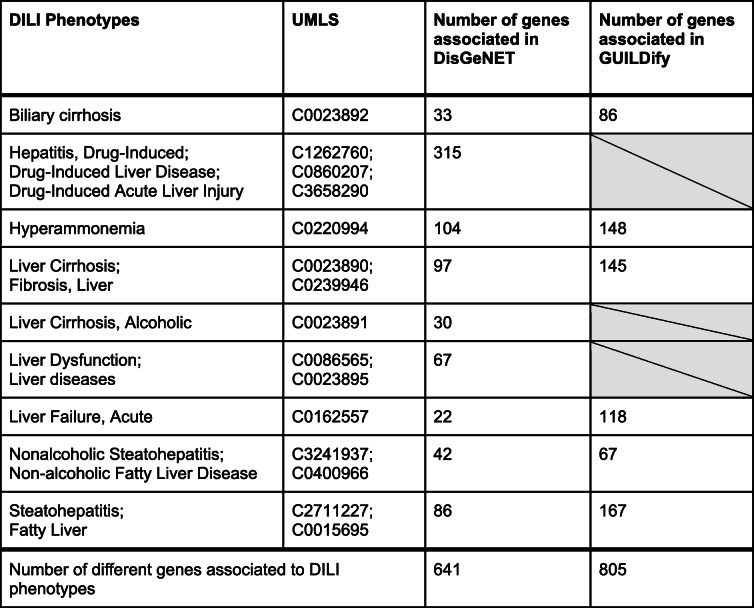
List of manually selected phenotypes related with DILI. The selected phenotypes were required to have 10 gene associations or more. The genetically redundant phenotypes have been merged in the same term. The empty cells correspond to phenotypes for which the expansion through the network using GUILDify was not functionally coherent

#### Drug chemical structure

The chemical structures of the drugs considered in the study were provided by the CAMDA challenge in the form of Simplified molecular-input line-entry system (SMILES) string. In order to use this type of data, we calculated the similarity between all compounds, creating a matrix of chemical similarity. Specifically, we used the R package *RxnSim* [24] to calculate the similarity matrix using the Tanimoto distance [25]. We used the function *ms.compute.sim.matrix* (default parameters), which identifies the fingerprints of the SMILES and computes the fingerprint similarity between pairs of SMILES. The full list of SMILES is provided in Supplementary Table 2, and the matrix of Tanimoto similarity between SMILES in Supplementary Table 3.

#### Drug-target association

The targets of the compounds considered in the study were retrieved from three different databases: DGIdb [26], HitPick [27] and SEA [28]. DGIdb gathers validated drug targets, whereas HitPick and SEA additionally provide predicted targets based on chemical similarity. We used the names of the drugs to retrieve the drug-protein associations from DGIdb, whereas the SMILES strings were used in the case of HitPick and SEA web servers. Any drug-protein pair that had been provided either by the database or predicted to interact by the web servers were included among the drug-target associations. This implies that there are no differences between validated and predicted targets. However, this allowed us to increase the number of input drugs and extended the potential recall of our method. After collecting all targets, a matrix was created with all the drugs in rows and all the target proteins in columns. The cells of the matrix had values 1 (if the drug targeted the protein) and 0 (otherwise). There are three drugs from the DILIrank dataset (alaproclate, fluvastatin and tenofovir) and two drugs from the independent hold-out test dataset (entecavir and vinorelbine) without any targets in these databases. These drugs have not been used neither for training nor for testing when using drug targets as features. The full list of drug-target associations is provided in Supplementary Table 4.

### Prediction pipeline

We created a supervised machine learning pipeline (Supplementary Fig. 1) to generate predictions using the features described in Table 1. The pipeline was implemented using the R package *caret* [29]. Briefly, we used two classifiers: the random forest classifier and the gradient boosting machine. We limited the number of classifiers because CAMDA had a limited number of independent hold-out test trials, and we tested many different features. Thus, we focused on two tree-based ensemble methods that have been widely employed in previous research [30–32].

We created a balanced dataset containing the 30% of the data for testing and the rest for training. The original dataset is comprised of 124 drugs labelled as DILI (37 as Most-DILI-Concern and 87 as Less-DILI-Concern) and 51 labelled as no DILI. To create a balanced testing dataset, as there were less drugs labelled as no DILI, we randomly picked the 30% of the 51 no DILI drugs (15 drugs), and the same number of DILI drugs, maintaining the ratio of Most-DILI-Concern (29.8%) and Less-DILI-Concern (70.2%): 4 Most-DILI-Concern drugs (the 29.8% of 15) and 11 Less-DILI-Concern drugs (the 70.2% of 15). The rest of the drugs (109 DILI drugs and 36 no DILI drugs) were used for creating multiple training datasets. In order to have balanced training datasets, while at the same time, to cover as many DILI drugs as possible, we created 10 different training datasets. All of them have the same 36 no DILI drugs (corresponding to the 70% of the initial 51 drugs), but each of the training dataset has a different subset of DILI drugs. Accordingly, among the 109 DILI drugs, we picked randomly 11 Most-DILI-Concern drugs (29.8% of 36) and 25 Less-DILI-Concern (70.2% of 36) (see Supplementary Fig. 1 for a schematic representation of the procedure, and Supplementary Table 5 for a detailed list of the number of drugs used in each step).

The 10 training datasets were used to train 10 different models. For each model, the hyperparameters of the machine learning classifier were tuned using the functions *trainControl* and *train* from the R package *caret* [29]. Specifically, we used a 10-fold cross-validation approach, allowing resampling of the training set to avoid overfitting. The *train* function automatically tests different models using several combinations of hyperparameters and selects the model with higher accuracy. The 10 fitted models were evaluated using the testing dataset, obtaining a series of measures (accuracy, precision, sensitivity, specificity, F1-score, MCC) that indicate the quality of the model. Lastly, the testing set predictions of the 10 models were used as features to train a random forest classifier that combined them into a final model. The final model was used to classify the drugs of the independent hold-out test dataset into DILI drugs and non-DILI drugs.

## Results

### L1000 connectivity map data hints at transcriptomic heterogeneity of DILI compounds

CMap collects gene expression signatures obtained from cell lines upon treatments with different drug concentrations and durations. The treatment dose ranges from the drug’s reported effective concentration, if known, to a relatively high concentration of 10 μM or more, often adopted in high-throughput cell based screens [33]. In order to include perturbations possibly leading to adversities or able to challenge cells adaptive mechanisms, we decided to focus on drugs tested at the highest concentration and for the longest treatment duration (i.e. high coverage, high dose, and long treatments). Therefore, we focused only on the samples treated at 10 μM dose and at least for 24 h. Furthermore, as DILI phenotypes are mainly originated and affecting the liver, we decided to study only those sets collected from the cell line “Primary Human Hepatocytes” (PHH), as to date, it is the most specific in vitro cellular model for liver. This produced a final set of samples with a single dose-time point from 51, 87, and 37 drugs annotated as No-DILI-Concern, Less-DILI-Concern, and Most-DILI-Concern, respectively.

**Fig. 1:**
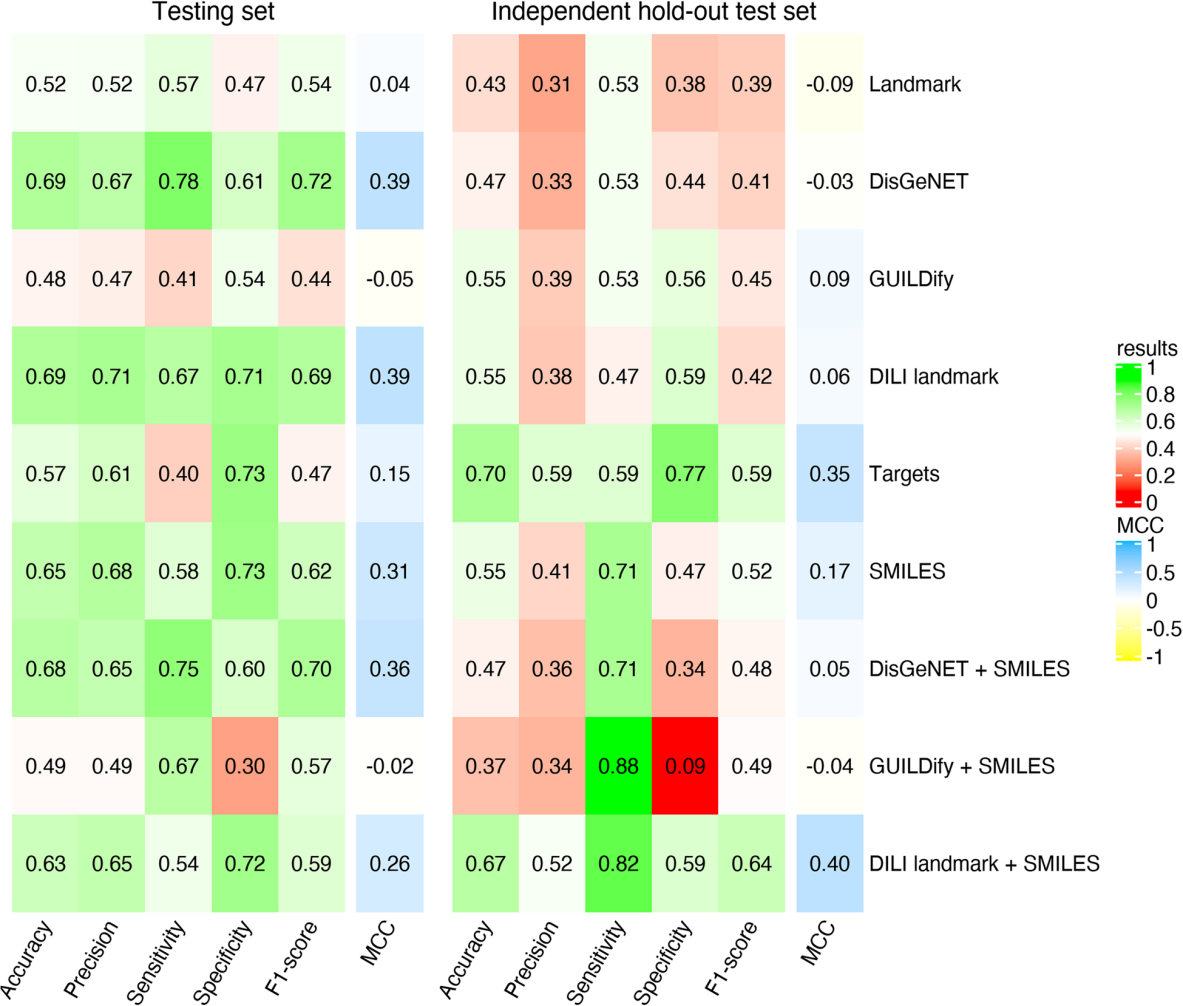
Results of the Classifiers in the testing set and the independent hold-out test set. The machine learning algorithm used was a Random Forest. The features that were used in the models of DisGeNET, GUILDify, DisGeNET+SMILES and GUILDify+SMILES are only from the phenotype “Biliary cirrhosis” (C0023892). The results of using different phenotypes are given in the Fig. 3. The results for gradient boosting machine classifier are given in the Supplementary Fig. 8

**Fig. 2:**
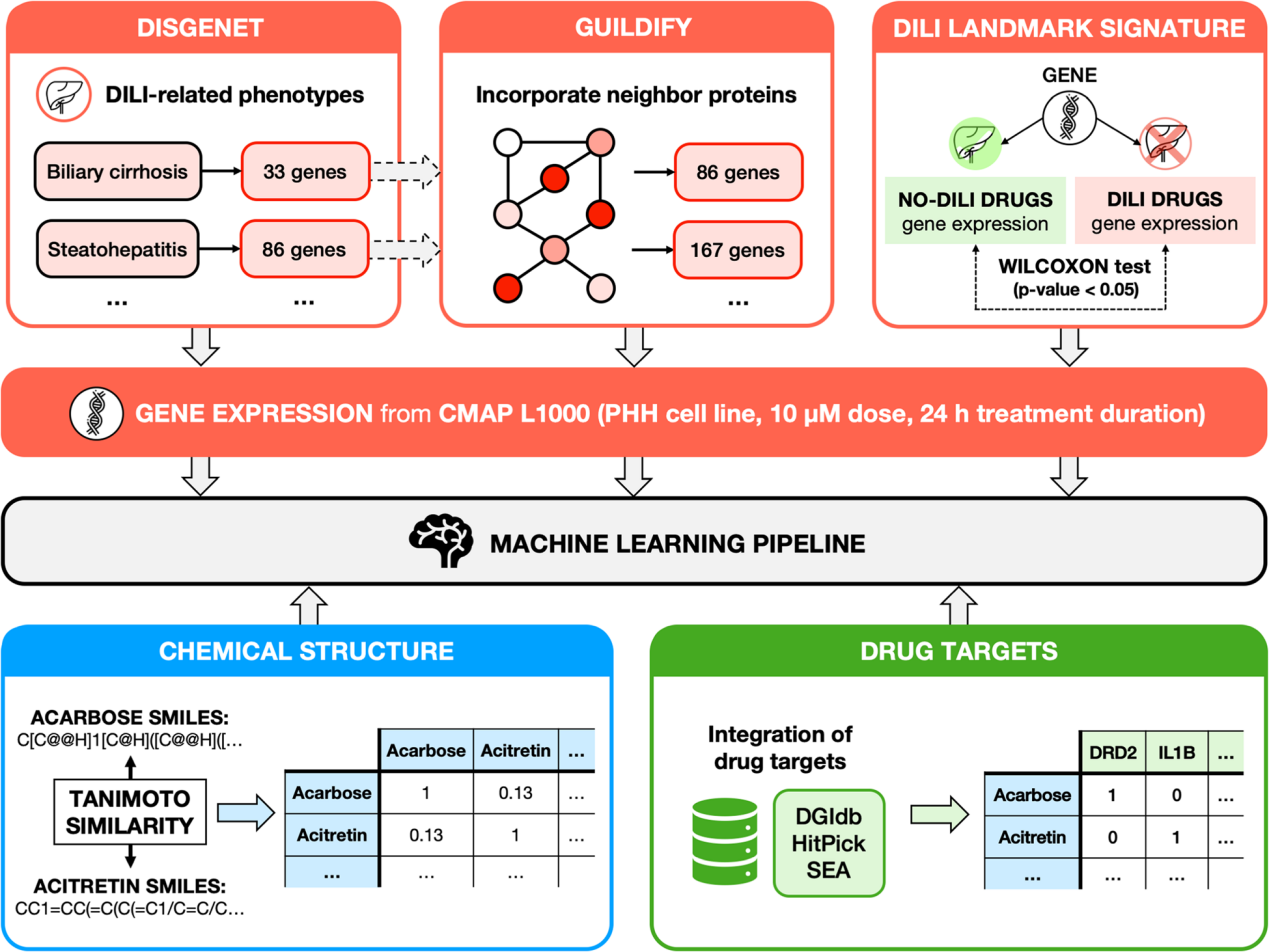
General scheme of the processing of the different features

As an initial exploratory analysis of the training data set, we analyzed the transcriptional response of the different drugs using k-nearest neighbor clustering algorithm (k = 3,4,5) (Supplementary Fig. 2). In the plot, we cannot distinguish the different groups of drugs based uniquely on gene expression and thus a more specific gene signature is needed. Indeed, we applied the landmark genes signature as a feature for a machine learning algorithm (as described in the Methods section) obtaining a mean accuracy of 52% in the testing set and 43% in the independent hold-out test set (Fig. 1). Perhaps more relevant are the low values of MCC (0.04 in the testing set and − 0.09 in the independent hold-out test set), which indicates that the level of expression of landmark genes (978) from CMap is not a predictor of DILI. In view of these results, we decided to look for alternative chemical structure, gene and phenotype based signatures. In the following sections, we explain the different strategies we developed to characterize DILI (Fig. 2).

### Using phenotype-gene associations highlights potential connections between DILI, cirrhosis and drug induced hepatitis

To characterize genes involved in DILI that could be used as a gene signature in the classifier, we searched for specific genes associated with DILI looking into phenotype-genotype data. These data contain genes that have been described as associated to the pathophysiology or etiology of DILI, and therefore represent a suitable source to develop a list of genes representative of DILI. We manually curated a list of phenotypes closely related with DILI and identified the genes associated with these phenotypes using the DisGeNET database v6.0 [17] (Table 2, Supplementary Table 1). Although we might expect them to be genetically similar, the overlap of genes between the different DILI phenotypes is very small (Supplementary Fig. 3). This fact reflects the diversity of the phenotypes considered and the challenge associated to predict DILI based solely on gene expression.

**Fig. 3:**
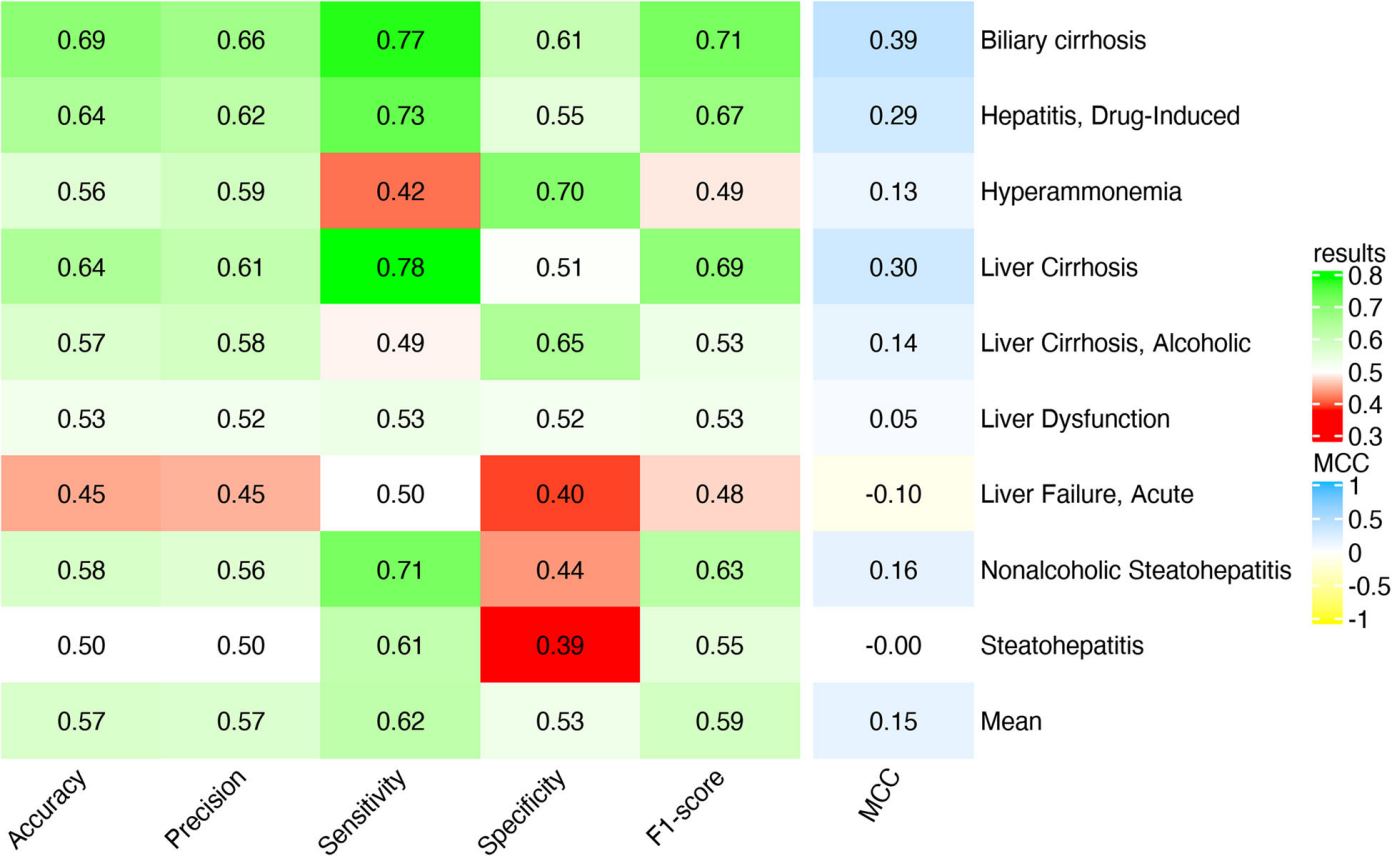
Results of the classifier based on gene sets from DisGeNET DILI phenotypes in the testing set. The machine learning algorithm used was Random Forest. Each row corresponds to the mean performance of 10 models trained using the PHH gene expression of the genes associated to each DILI phenotype. The “Mean” row corresponds to the average performance of each metric for all the phenotypes

Once defined the set of genes for the different DILI-related phenotypes as annotated in DisGeNET, we retrieved their gene expression data from the CMap L1000 Assay Platform. For each DILI-related phenotype, we trained an independent machine learning model using the expression levels of their genes as features. The average accuracy obtained for the models of all DILI-related phenotypes is 57% in the testing set. This means that for some specific phenotypes the accuracy was higher than 57%. Therefore, we inspected the results for all phenotypes separately, observing those with higher accuracy than others (Fig. 3). The phenotypes “Biliary cirrhosis”, “Hepatitis, Drug-Induced” and “Liver cirrhosis” stand out for having an accuracy between 64 and 69% and values of precision, sensitivity and specificity above 50%, and MCC above 0.3. It is worth noting that “Biliary cirrhosis” is the phenotype less genetically similar to the rest, i.e. the lowest number of shared genes (Supplementary Fig. 3) yielding the best results of prediction. Among the genes associated with these phenotypes, some of them have been associated to hepatotoxicity by a previous study of *Peng* et al. *(2019)* [34], where 145 hepatotoxicity-related genes were identified. “Biliary cirrhosis” contains 5 hepatotoxicity-associated genes, “Hepatitis, Drug-Induced” has 27 and “Liver cirrhosis” 25 (Supplementary Table 6).

### Incorporating protein-protein interactions to find a DILI signature does not improve the results of phenotype-gene associations

Our current knowledge of genotype-phenotype associations is still incomplete and therefore we might miss relevant genes associated to DILI. It has been demonstrated that the products of disease-associated genes tend to be highly connected in the protein-protein interaction network, forming the so-called disease modules [35, 36]. Based on this fact, network-based prioritization methods exploiting the topology of the protein-protein interactions network have been successfully applied to discover and prioritize novel disease-gene associations [37].

Using the network-based prioritization web server GUILDify [38], we extended the current knowledge of disease-associated genes obtained from DisGeNET (see above in the previous section). GUILDify uses the genes associated with DILI-related phenotypes as seeds for an algorithm that scores the proteins of the protein-protein interaction network based on their topological closeness with the seeds. Then, it selects the top-ranking genes using a functional-coherency-based cut-off: non-seed genes are iteratively included in the top-ranking set provided that they maintain the functional coherency of the seed genes (they are involved in similar biological functions). The numbers of the new associations with the DILI-related phenotypes are listed in Table 2.

After obtaining the new list of phenotype-gene associations, we retrieved their gene expression data from the CMap L1000 Assay Platform as shown before (i.e. PHH cell line with 10 μM dose and treatment duration of 24 h) and used the expression level of these genes as input feature to the machine learning classifier. As shown in Fig. 1 the predictive capacity of the classifiers in the training set dropped with regards to the approach described in **Section 2**, obtaining similar values to that of when using the 978 landmark genes albeit with a slightly higher specificity (see results by phenotype at Supplementary Fig. 4).

### Differential comparison of gene expression does not produce a robust DILI signature

To investigate the extend the transcriptomics data on drugs with known DILI status could be used to extract a DILI gene signature, we retrieved the normalized differential expression data of the genes in PHH cell line (10 μM dose and treatment duration of 24 h). For each gene, we checked whether the expression values were significantly different between DILI and No-DILI-Concern drugs. Therefore, for each landmark gene, we applied a two-sided Wilcoxon test, a non-parametric test comparing the expression of the gene in the samples of DILI-Concern drugs and No-DILI-Concern drugs. We selected the genes with a *P*-value lower than 0.05, obtaining a gene signature composed of 66 genes (referred from now on as DILI landmark gene signature) (see Supplementary Table 7). We chose to use marginal *P*-values, focusing on the ranking of genes and aiming to capture the broad transcriptomic DILI signal.

**Fig. 4:**
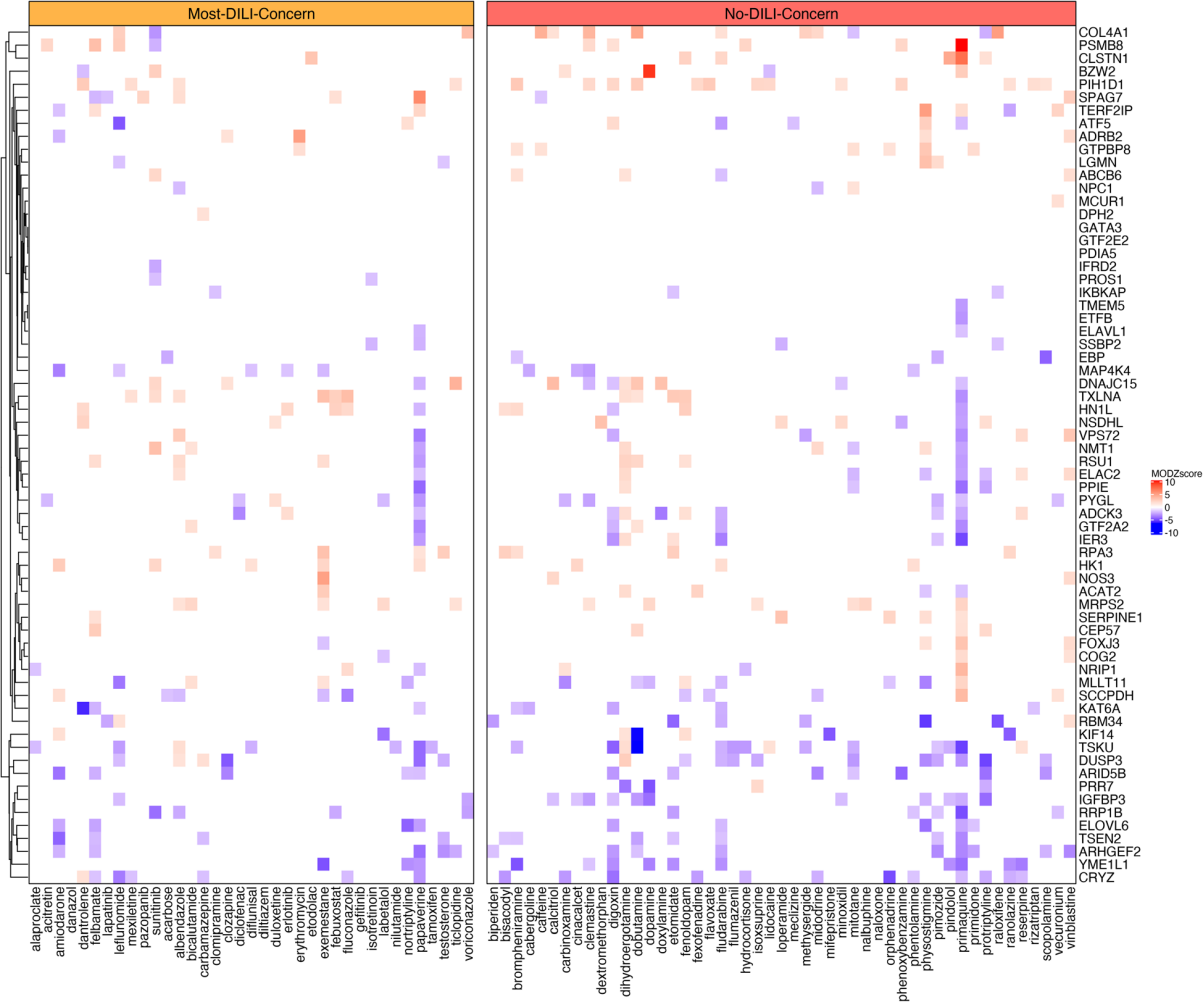
Transcriptomics signatures of the DILI landmark genes. Gene expression (as Moderated Z score) of the DILI landmark genes (selected using a two-sided Wilcoxon rank sum test, *P* < 0.05) in PHH cells, for Most-DILI-Concern and No-DILI-Concern drugs. The gene expression lower than |1.5| is colored white

Consistent with the known heterogeneity of transcriptomics response in hepatotoxicity, the genes in the identified signature were typically perturbed only in a small subset of the samples, failing to represent a common response that could be explained by gene expression changes (Fig. 4). However, while the gene expression of the 1000 landmark genes yielded an accuracy of 52% (43% in the independent hold-out test set), using only the 66 selected genes increased the accuracy to a 69% (55% in the independent hold-out test set). The discrepancy between the testing and independent hold-out test sets can be attributed to the gene expression signature likely fitting to the underlying biology of the training set compounds rather than representing a generalization across all potential DILI compounds. We performed a functional enrichment analysis [39] using Gene Ontology to further investigate the biological processes of these genes.

The functional enrichment analysis of the 66 genes did not yield specific functions significantly associated with the genes. This indicates that, even though the selected genes improve the capacity of the classifiers to predict DILI-causing drugs in comparison with using all the landmark genes, they are not related with specific biological processes. Additionally, we compared these 66 genes to the 145 identified by *Peng* et al. *(2019)* [34] as associated to hepatotoxicity (Supplementary Table 6). Only 4 genes were highlighted as hepatotoxic in *Peng* et al’s study. Therefore, although the approach succeeded at improving the predictive capacity of the classifiers, the results could lead to overfitting by the available data, as the gene signature is not related to any specific biological function.

### The use of chemical structure and drug-target associations increases the prediction accuracy

Besides using gene expression data, we investigated the incorporation of orthogonal information derived from chemical structure of the drug and its targets. The chemical structure and the molecular descriptors of the drugs have already been used in machine learning models, showing a fair predictive capacity [10]. Here, we used the Tanimoto similarity between the molecular fingerprints of all the drugs in the dataset. First, we plotted the similarity between Most-DILI-Concern and No-DILI-Concern drugs in the dataset in a histogram (Supplementary Fig. 5). We observed that drugs of the same group did not have higher similarity among them than with other groups. Furthermore, No-DILI-Concern drugs have higher similarity between themselves (mean 0.24) than Most-DILI-Concern drugs (mean 0.18). This indicates that there is a considerable structure heterogeneity within the Most-DILI-Concern group of drugs, which complicates the prediction using chemical structure. But this also suggests that probably, when combining this feature with other types of features (i.e. transcriptomics), the prediction may improve.

Eventually, we obtained a higher prediction accuracy when combining chemical structure and CMap than when applying them separately (Fig. 1). When using solely chemical structure as feature for the machine learning classifiers the average accuracy was 65% (MCC of 0.31) in the test set and 55% (MCC of 0.17) in the independent hold-out test set. In contrast, when combining chemical structure with transcriptomics data, the prediction of the classifier in the independent dataset improved. This is remarkable when combining it with the DILI landmark gene signature derived from the nonparametric test: the average accuracy is maintained at 63% (MCC of 0.26) in the test set and increases to 67% (MCC of 0.40) in the independent hold-out test set (Fig. 1).

Next, we explored the use of drug-target associations as a feature to predict DILI. We considered any drug-protein pair that had been reported in DGIdb [26] or predicted to interact by HitPick [27] or SEA [28]. We integrated targets from these databases, creating a matrix containing drugs and target proteins (see Methods). We analyzed the percentage of DILI drugs, no-DILI drugs and drugs from the independent dataset associated to the targets in the matrix (see Supplementary Table 8 and Supplementary Fig. 6). We observed that some proteins are mostly targeted by one type of drug, hypothetically facilitating the classification of drugs. For instance, proteins such as CYP2C9 and CYP1A2, that are associated with a higher proportion of DILI drugs than to no-DILI drugs, have been previously associated to hepatotoxic effects [40, 41]. Thus, we used the matrix as a feature for the machine learning classifiers, obtaining an accuracy of 57% (MCC of 0.15) in the testing set and 70% (MCC of 0.35) in the independent hold-out test set (Fig. 1). The increase of accuracy in the independent dataset is explained by the high specificity (i.e. no-DILI drugs are predicted correctly) in contrast with the low sensitivity (i.e. DILI drugs are not predicted correctly).

### Hepatocyte cell lines provide a better context for DILI prediction than using combined expression from different cell lines

In the previous sections, when using CMap gene expression data, we selected only the samples from the PHH cell line with 10 μM dose and treatment duration of 24 h. We focused on the drug response in liver cells. However, the data of CMap tends to have a high variation of expression between samples even for the same gene. Therefore, to avoid biases caused by the use of unrelated samples, we experimented using only the top correlated samples for each drug. This consists in computing the correlation between all the samples exposed to a drug (even if they are from different cell lines, doses and treatment durations) and selecting the ones that are more correlated between themselves. We selected the pairs of samples from different cell lines that have a Pearson correlation above 0.5, or otherwise we kept the pair that was more correlated. To use a correlation threshold of 0.5 guarantees that the expression of the samples selected is consistent enough across several cell lines. Once the correlated samples are selected, we use the median gene expression as feature. Although the approach was theoretically promising, the prediction accuracies with the use of correlated samples are generally worse than using specific conditions, obtaining MCC values ranging from − 0.12 to 0.21 (see Supplementary Fig. 7). This indicates that we are still getting noise from correlated samples and that, even if there are some samples that could be less reliable, the use of specific liver conditions in gene expression seems to be the best approach for the prediction of DILI-Concern drugs.

## Discussion

In this work, we aim to predict DILI applying machine learning algorithms using a range of orthogonal types of data as input features. Indeed, we explored the use of gene expression data from different sets of selected genes (i.e. landmark, DisGeNET and GUILDify sets) alone and in combination with drug-centric information in the form of structural similarity (Tanimoto scores) and protein targets (see Table 1 for a brief description of the features). Furthermore, we observed that the DILI landmark gene signature identified by a non-parametric test (Wilcoxon test) of differential expression between DILI and no-DILI samples from PHH cell line constituted a better feature set than the whole landmark genes in CMap.

The genes in the identified DILI landmark gene signature were typically perturbed only in a small subset of the samples, failing to represent a response that could solely be explained by gene expression changes (Fig. 4). This finding is consistent with the known heterogeneity of transcriptomics response in hepatotoxicity [42]. Also, it could be related with the diversity of outcomes of the different compounds (i.e. acute, chronic or idiosyncratic reactions). Nevertheless, as we were using data from the training set to obtain the signature, the results could lead to overfitting, which would explain why the accuracy of the prediction in the independent hold-out test worsened. Moreover, the drugs of the independent hold-out test set were originally flagged as ambiguous and for this reason are probably a more challenging set to classify. Also, the independent hold-out test could be unbalanced, worsening the results despite the classifier being trained on a balanced dataset.

We also took advantage of functional information of the genes involved in drug response, and evaluated gene expression related to liver phenotypes involved in drug response using DisGeNET resource [17]. In the same way as before, limiting the number of genes to a specific signature (the genes associated to a DisGeNET phenotype) also constituted a better feature set than the whole landmark genes in CMap, but still failed to represent the whole response. The best accuracy was achieved by the phenotype “Biliary Cirrhosis”, which is one of the final stages of DILI. Since we used the data from the highest dose and time point, it makes sense that extreme phenotypes related with liver cirrhosis are better predictors. Perhaps, the biliary component is also important for the predictor. For further studies, it would be interesting to focus on lower doses in order to capture earlier events and not the final extreme phenotype. It is also important to remark that the gene expression signatures come from an in vitro model (primary cells, but still with the limitations of 2D, dedifferentiation, etc.). As we applied gene signatures derived from human data, this could have affected the results.

Additionally, we expanded the phenotype-gene associations retrieved from DisGeNET incorporating protein-protein interactions data from GUILDify. By applying GUILDify we could expand the number of genes associated with DILI-related phenotypes by incorporating those connected by the underlying protein interactome. Surprisingly, the quality of the prediction when adding protein interactions decreased with respect to using solely phenotype-gene associations. Our hypothesis is that when expanding the number of genes using GUILDify, (i.e. obtaining larger gene signatures), the intrinsic data noise from the CMap dataset is increased as well, hence hampering the prediction. Still, we think that using protein-protein interactions to extend our information on DILI targets and hepatotoxicity-associated genes without using gene expression data could be an interesting feature to explore in the future.

After working with transcriptomics data from CMap, we observed variability of the results depending on the pre-processing of the samples. We tried two different strategies that led to different results: (i) focusing on samples from a unique cell line and dose-time point for each drug, and (ii) selecting the most correlated samples for each drug. This is by no means comprehensive and various possible strategies such as using other cell lines and dose-time points, or discarding the samples with low correlation between replicas (‘distil_cc_q75’ < 0.2) and selecting the sample with highest transcriptional activity score [43] could be investigated further.

When focusing on samples from a unique cell line and dose-time point, we decided to use the highest concentration and the longest treatment duration. In this way, we were including perturbations possibly leading to adversities for the adaptive mechanisms of the cells. We acknowledge that focusing on increased exposure of the drug to characterize DILI is a relatively strong assumption as there could be certain compensatory mechanisms kicking in after a while depending on the specific compound and cell line. Nevertheless, we think that employing the highest dose at the longest time of exposure is likely to be a fair representation of the effect of DILI in the cells after the administration of the drug.

Apart from the limitations inherent in the CMap dataset, we detected: (i) an important genetic diversity between the diverse DILI-related phenotypes from DisGeNET (Supplementary Fig. 3), and (ii) a great structural diversity between the drugs reported as DILI-Concern (Supplementary Fig. 5). Both aspects hamper the prediction of DILI-Concern drugs when using transcriptomics or structural features separately and encouraged us to use and combine other sources of information.

When considering both transcriptomics and structural features together, we observed a similar predictive power of the classifiers, but a general increase when validating the classifiers with an independent dataset (Fig. 1). The most accurate classifier was generated by the Random Forest algorithm using a combination of features that included the chemical similarity of drugs (Tanimoto coefficient calculated using SMILES) and gene expression from the landmark genes selected with a non-parametric test (DILI Landmark + SMILES). Under a benchmark scenario, the classifier was able to separate DILI-Concern drugs better than No-DILI-Concern drugs (accuracy 63%, sensitivity 54% and specificity 72%). Furthermore, on the independent dataset of ambiguous-DILI drugs re-labelled by the FDA, it reached an accuracy of 67%, the second highest among the different classifiers. In the future, it would be interesting to use the drug structures directly as features (without using their similarities) and to combine them with the other types of features, as there might be critical information within the actual molecular details of the drugs.

Lastly, we explored if the use of drug-target associations could be useful to predict DILI-causing drugs. The results showed that the targets of most DILI drugs were related with hepatoxicity (Supplementary Fig. 6). The use of drug-target associations as a feature produced an accuracy of 57% in the testing set and 70% in the independent dataset. The observed accuracy on the independent dataset is in line with 72.5% sensitivity and 72.7% specificity of the computational model developed by *Zhang* et al. as well as with the 70.9% accuracy obtained by *Hong* et al. on the bootstrapped data set, highlighting the current limitations in predicting drug induced injury [8, 9].

When comparing the results with the publications of the previous CAMDA 2018 edition [12, 13], we still do not observe a clear improvement on the prediction of DILI. Although the data provided is much more extensive, including gene expression data from more cell lines, the gold standard is still very reduced and unbalanced. The results in terms of accuracy in the training set are very similar to the ones obtained by *Sumsion* et al. [12], but worse when looking at the independent hold-out test. This is probably due to the fact that the current independent dataset is based on “Ambiguous-DILI” drugs, making the task more challenging. In terms of MCC values, our results (ranging from − 0.05 to 0.39 in cross-validation and − 0.09 to 0.40 in independent hold-out test) are slightly better than the ones reported in *Chierici* et al. [13] (ranging from − 0.04 to 0.21 in cross-validation and − 0.16 to 0.11 in the independent hold-out test set). Still, while the two published approaches of the previous edition were more focused on testing and optimizing different types of machine learning classifiers, our study focused on evaluating different types of features and searching a specific DILI gene signature. Therefore, the point of view of our work has been very different and complement previous approaches.

Overall, our results pointed to a mild variation on the accuracies depending on the samples included in the training data as well as the feature set used in building the classifiers, which we attribute to various factors. First, the training data is limited to dozens of compounds with known hepatotoxicity annotation, and these are too few to get a robust classifier. Second, most compounds show a toxic effect based on the dosage (and are otherwise no-DILI), thus a global predictor categorizing drugs as simply DILI vs no-DILI might not be realistic. And, third, there is substantial heterogeneity in the transcriptomics data from CMap. There is also variation between the results of the testing set and the independent hold-out test set, that could be caused by the latter being unbalanced (as the labels remain hidden). Still, the variation between the machine learning algorithms (random forest vs gradient boosting machine) is not appreciable in most cases (see results for gradient boosting machine in Supplementary Figs. 8–9). This indicates that even though the classifiers are different, the results are consistent because they depend on the data rather than on the algorithms. Still, future work would be required to experimentally validate the predictions of these models.

## Conclusions

In this study, we developed an ensemble learning approach to investigate the mechanism of the drugs that cause DILI. We experimented with gene expression data from the CMap L1000 dataset both alone and in combination with other types of feature (chemical structure, drug targets). We observed that selecting a specific gene signature either using phenotype-gene associations data (DisGeNET) or a non-parametric test (Wilcoxon test) of differential expression between DILI and no-DILI samples constituted a better feature than the whole landmark genes in CMap. However, the accuracy of the best performing classifier is around the 70% mark (minimum 63%, maximum 76%), stating the limitations of predicting DILI. The results are very similar to previous publications [8–10, 12]. Additionally, we used the comparison of chemical structures as a feature to predict DILI-causing drugs, though this did not improve the accuracy substantially. When comparing the chemical structures of the drugs with the same DILI-Concern classification, we observed a large structural diversity among the DILI-Concern groups, reflected in their dissimilarity of structure. This may explain the limited accuracy prediction based on chemical structure. Combining transcriptomics data and chemical structure did not improve the accuracy of the prediction in the testing set, although this was improved in the independent hold-out test set. Specifically, the combination of using a DILI associated gene signature and chemical structures produced results of accuracy around or less than 70%, but more robust when they were validated with the independent hold-out test set. We also used drug-target associations as feature, obtaining 57% of accuracy in the testing set that improved to a 70% in the independent hold-out test set. Summarizing, the overarching goal of this work was to evaluate a range of descriptors to predict DILI employing two commonly used classifiers to predict DILI. We have shown the limitations and advantages of different sets of data paving the way for future research in this field.

## Supplementary Information

**Additional file 1.**

**Additional file 2.**

**Additional file 3.**

**Additional file 4.**

**Additional file 5.**

**Additional file 6.**

**Additional file 7.**

**Additional file 8.**

**Additional file 9.**

## Data Availability

The code used during the current study is available in the following repository: Availability: https://github.com/structuralbioinformatics/CAMDA2019-DILI

## Abbreviations

DILI: Drug-induced liver injury
CAMDA: Critical assessment of massive data analysis
CMap: Connectivity map
SMILES: Simplified molecular-input line-entry system
PHH: Primary human hepatocytes
RF: Random forest
