# Supplementary material for: An ensemble learning approach for modeling the systems biology of drug-induced injury
Source: Biol Direct. 2021 Jan 12;16:5. doi: 10.1186/s13062-020-00288-x (PMC7805064; doi:10.1186/s13062-020-00288-x)
Supplement: Supplementary file 9 — Additional file 9. [file 13062_2020_288_MOESM9_ESM.pdf]

Supplementary material for:
An ensemble learning approach for modeling the systems biology of drug-induced injury

Joaquim Aguirre-Plans, Janet Piñero, Terezinha Souza, Giulia Callegaro, Steven J. Kunnen, Ferran Sanz, Narcis Fernandez-Fuentes, Laura I. Furlong, Baldo Oliva, Emre Guney

Supplementary Figure 1 2
Supplementary Figure 2 3
Supplementary Figure 3 4
Supplementary Figure 4 5
Supplementary Figure 5 6
Supplementary Figure 6 7
Supplementary Figure 7 8
Supplementary Figure 8 9
Supplementary Figure 9 10
Supplementary Table 1 11
Supplementary Table 2 12
Supplementary Table 3 13
Supplementary Table 4 14
Supplementary Table 5 15
Supplementary Table 6 16
Supplementary Table 7 17
Supplementary Table 8 18

Supplementary Figure 1

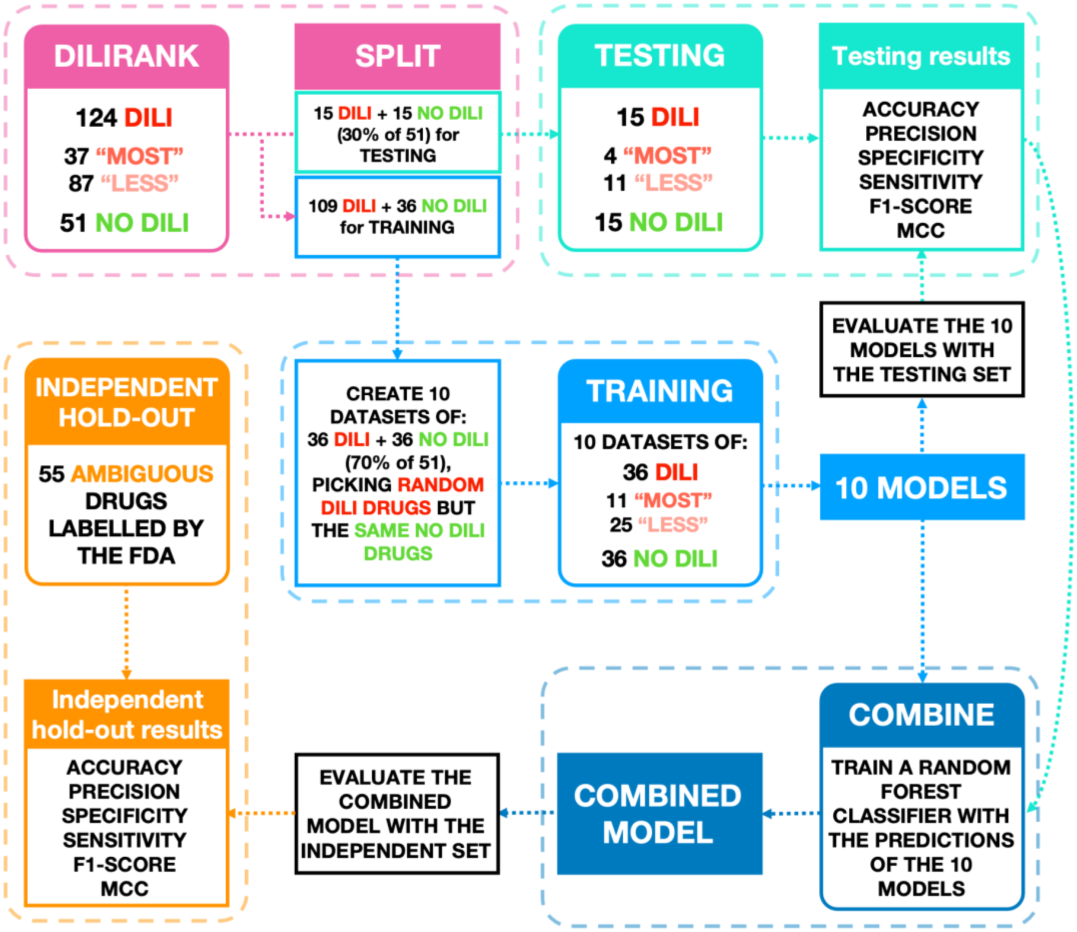

Supplementary Figure 1. Scheme of the machine learning pipeline. The DILIrank dataset is comprised of 124 drugs labelled as DILI (37 as Most-DILI-Concern and 87 as Less-DILI-Concern) and 51 labelled as no DILI. The dataset is randomly split into a balanced testing dataset made of 15 No-DILI-Concern drugs (30% of 51 drugs), and the same number of DILI drugs maintaining the ratio of Most-DILI-Concern (29.8%) and Less-DILI-Concern (70.2%): 4 Most-DILI-Concern drugs (the 29.8% of 15) and 11 Less-DILI-Concern drugs (the 70.2% of 15). The rest of the drugs (109 DILI-Concern drugs and 36 No-DILI-Concern drugs) is used to create 10 different balanced training datasets. For the 10 training datasets, we select the same 36 No-DILI-Concern drugs, but we pick randomly 36 drugs from the 109 DILI-Concern drugs: 11 Most-DILI-Concern drugs (29.8% of 36) and 25 Less-DILI-Concern (70.2% of 36). Using the 10 training datasets, we build 10 different models that are evaluated using the same testing dataset. The predictions of the 10 models are combined into a final model using a random forest algorithm. The final model is evaluated using the independent hold-out test dataset, comprising 55 drugs with hidden labels.

Supplementary Figure 2

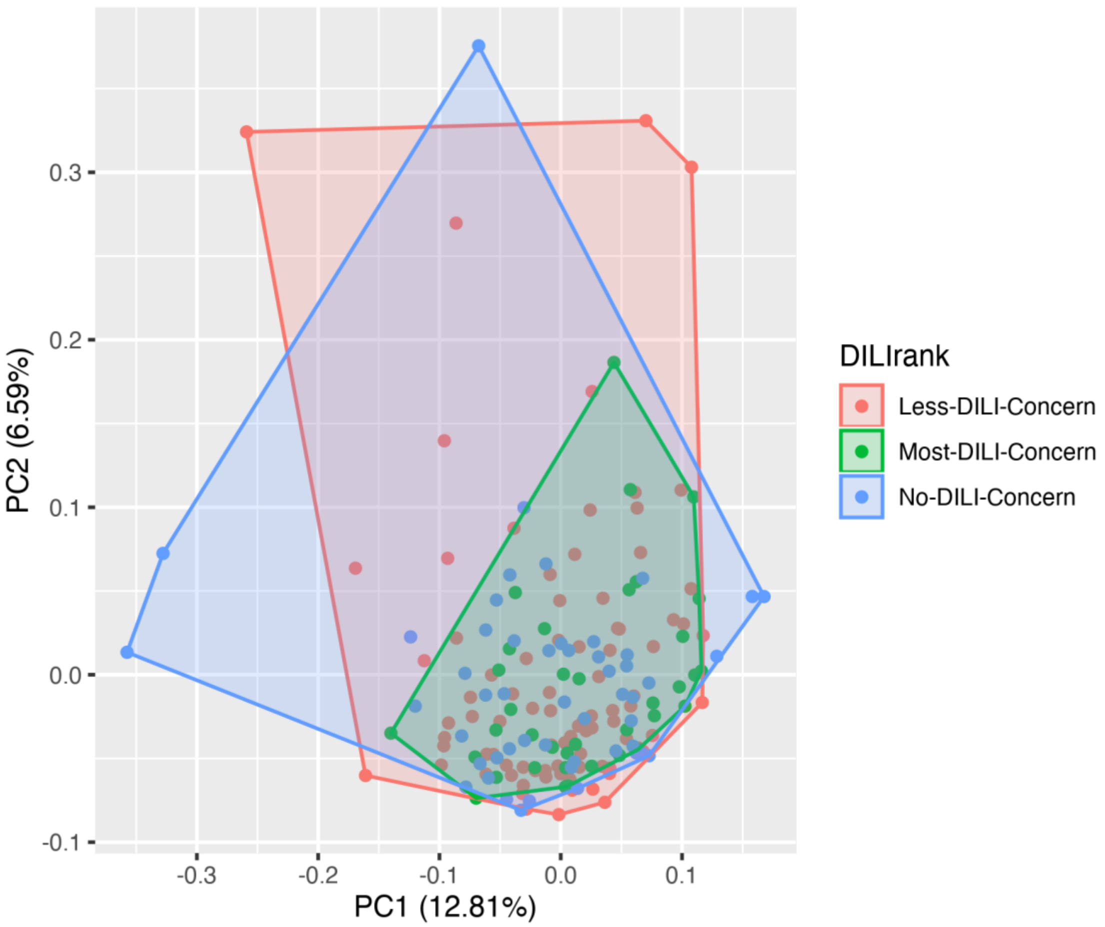

Supplementary Figure 2. Low dimensional representation of the gene expression of the training set compounds based on their transcriptomics profiles across samples of primary human hepatocyte (PHH) cell line and their DILI category.

Supplementary Figure 3

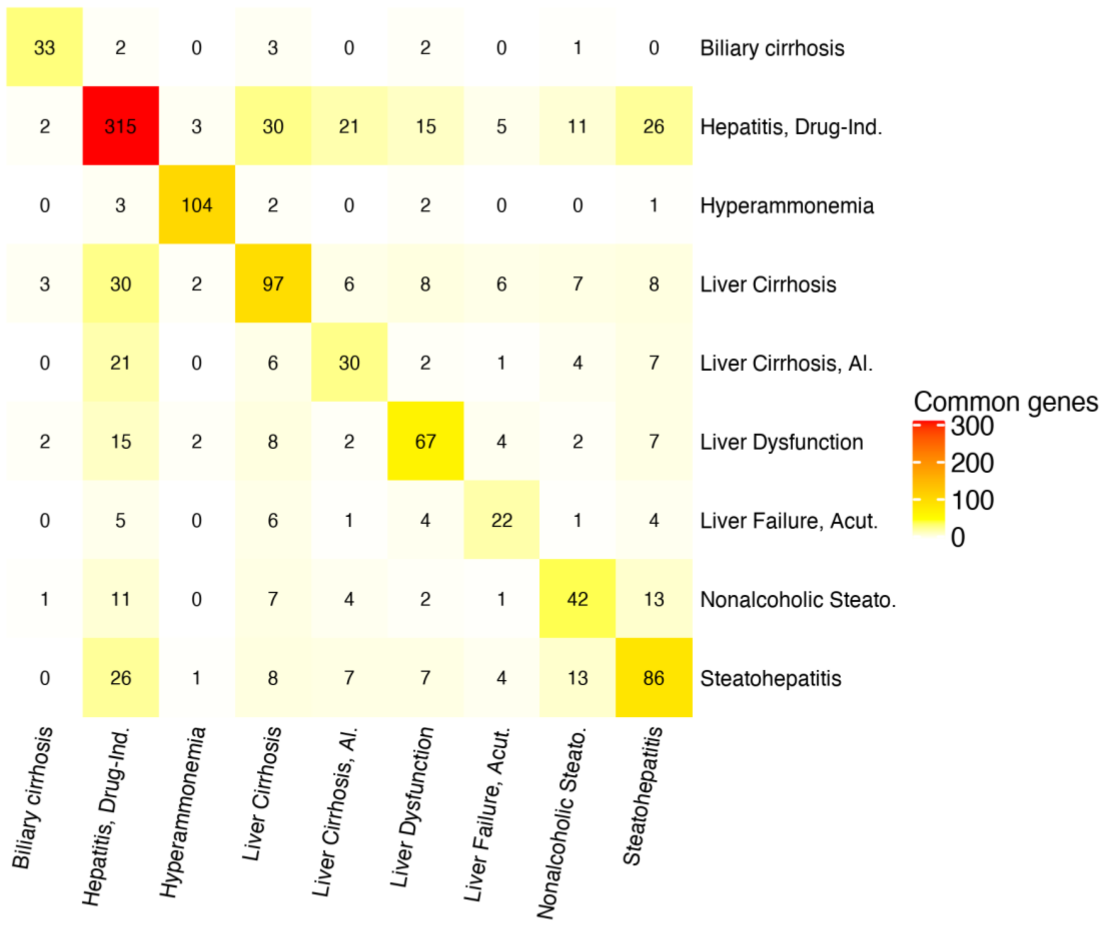

Supplementary Figure 3. Number of common genes between the Drug-Induced Liver Injury (DILI) phenotypes retrieved from DisGeNET.

Supplementary Figure 4

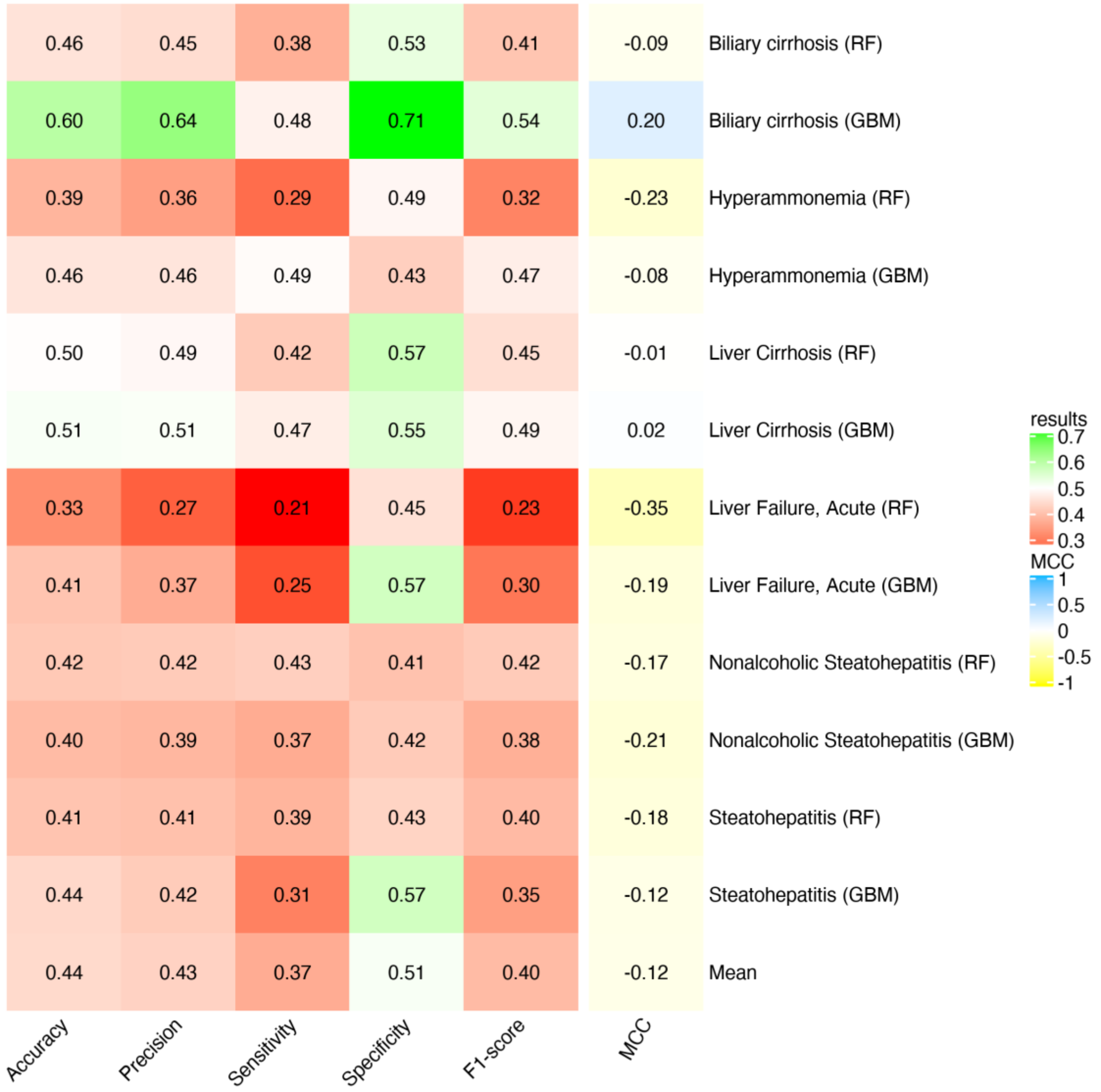

Supplementary Figure 4. Result of the classifiers based on gene sets from GUILDify DILI phenotypes in the testing set. The machine learning algorithms used are either Random Forest (RF) or Gradient Boosting Machine (GBM). Each row corresponds to the mean performance of 10 models trained using the PHH gene expression of the genes associated to each DILI phenotype. The “Mean” row corresponds to the average performance of each metric for all the phenotypes.

Supplementary Figure 5

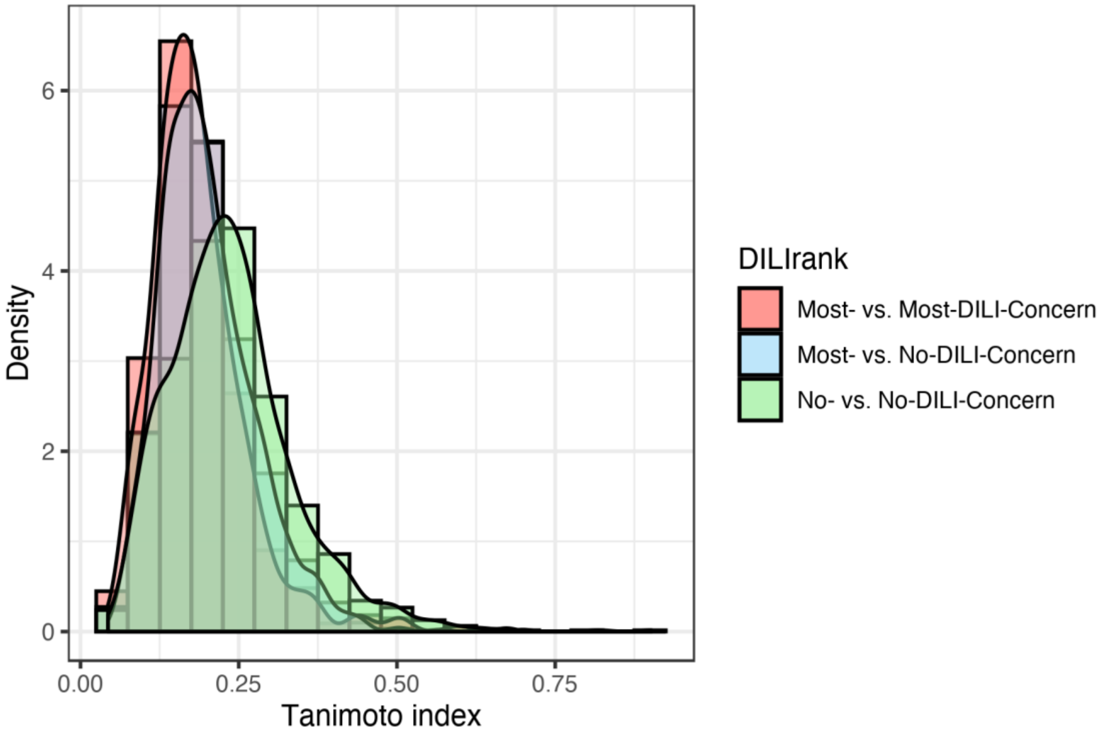

Supplementary Figure 5. Tanimoto similarity between the drugs in the DILI severity categories "Most-DILI-Concern" (Most-) and "No-DILI-Concern" (No-).

Supplementary Figure 6

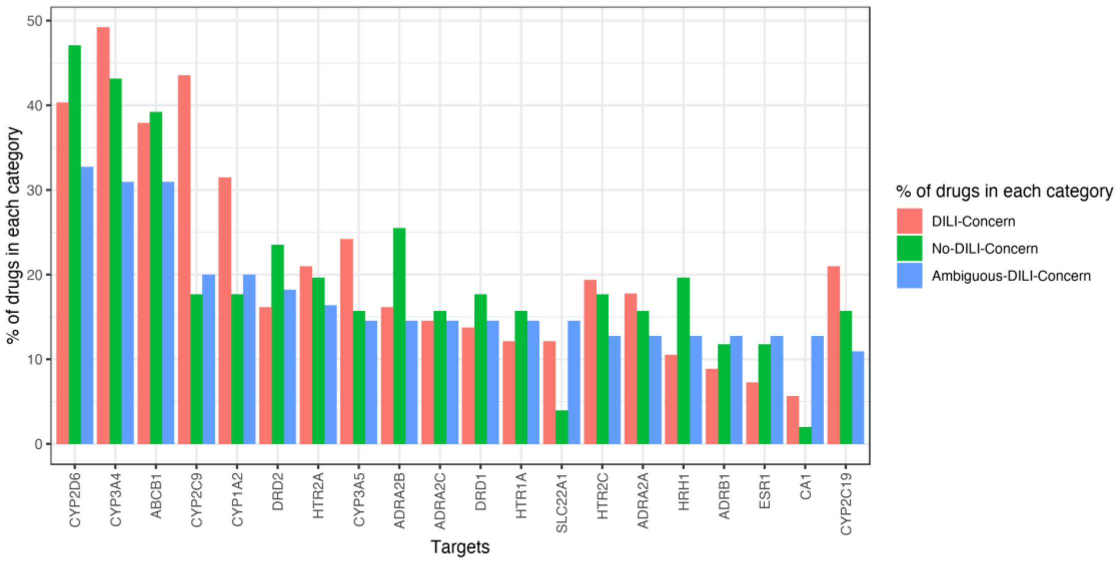

Supplementary Figure 6. Percentage of drugs in each DILIrank category that interact with a selection of 20 target proteins (Supplementary Table 8). Proteins in Supplementary Table 8 were selected as those targeted by the largest number of drugs in the independent hold-out test dataset.

Supplementary Figure 7

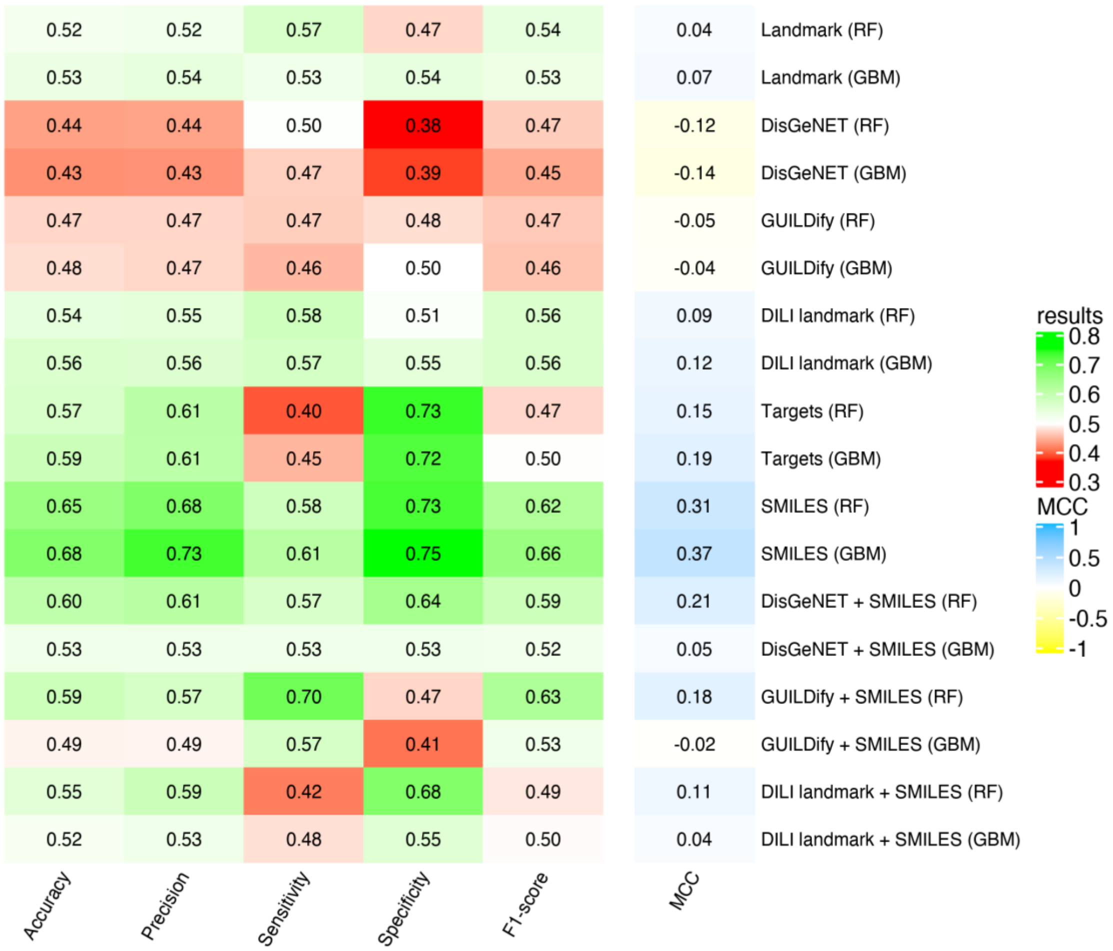

Supplementary Figure 7. Results of the Classifiers in the testing set when using transcriptomics features from the most correlated samples of each drug. The machine learning algorithms used are either Random Forest (RF) or Gradient Boosting Machine (GBM). Results of DisGeNET, GUILDify, DisGeNET+SMILES and GUILDify+SMILES are the mean of all the phenotypes' results.

Supplementary Figure 8

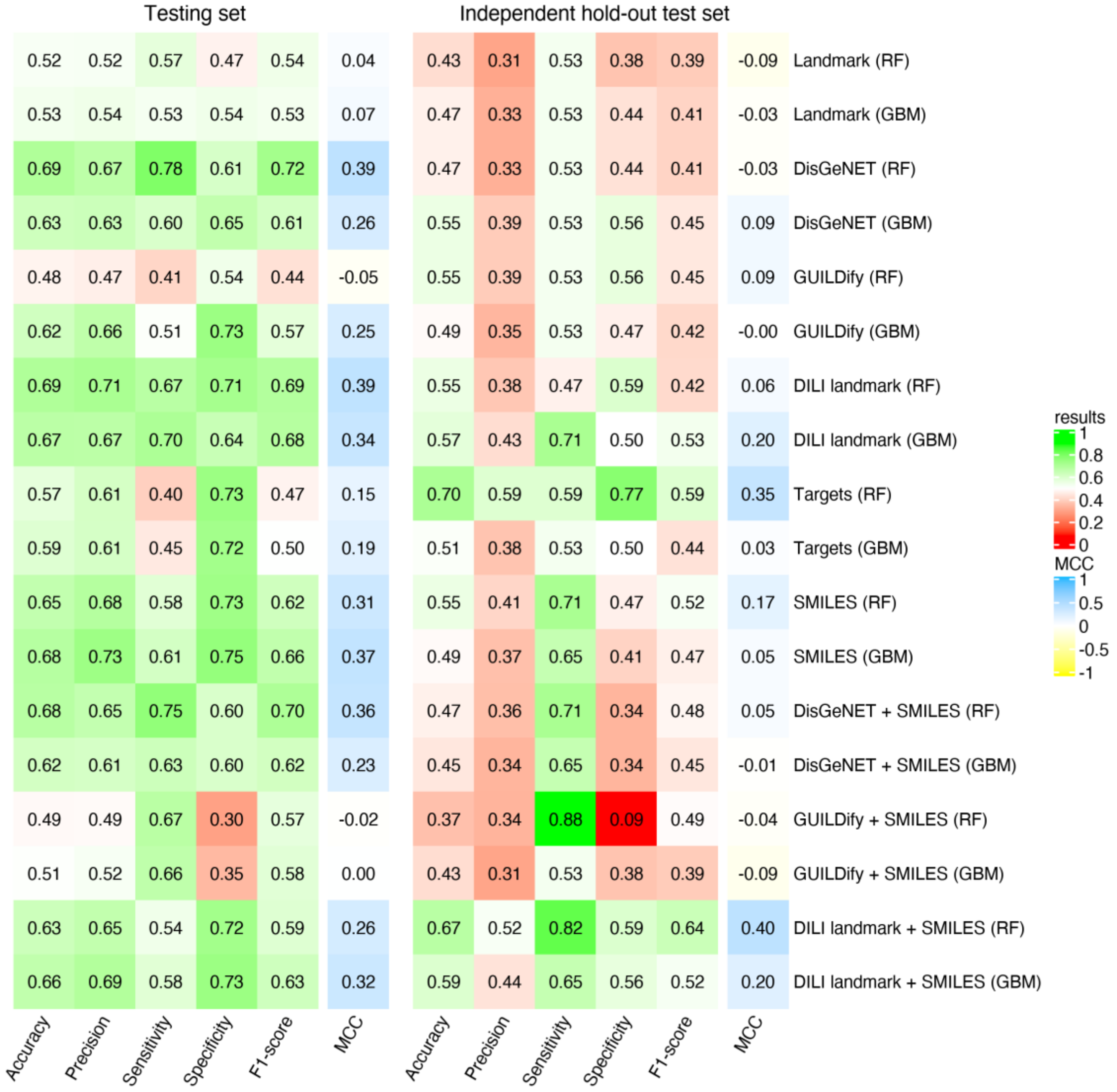

Supplementary Figure 8. Results of the classifiers in the testing set and the independent hold-out test set. The machine learning algorithms used are either Random Forest (RF) or Gradient Boosting Machine (GBM). Results of DisGeNET, GUILDify, DisGeNET+SMILES and GUILDify+SMILES are from the phenotype “Biliary cirrhosis” (C0023892).

Supplementary Figure 9

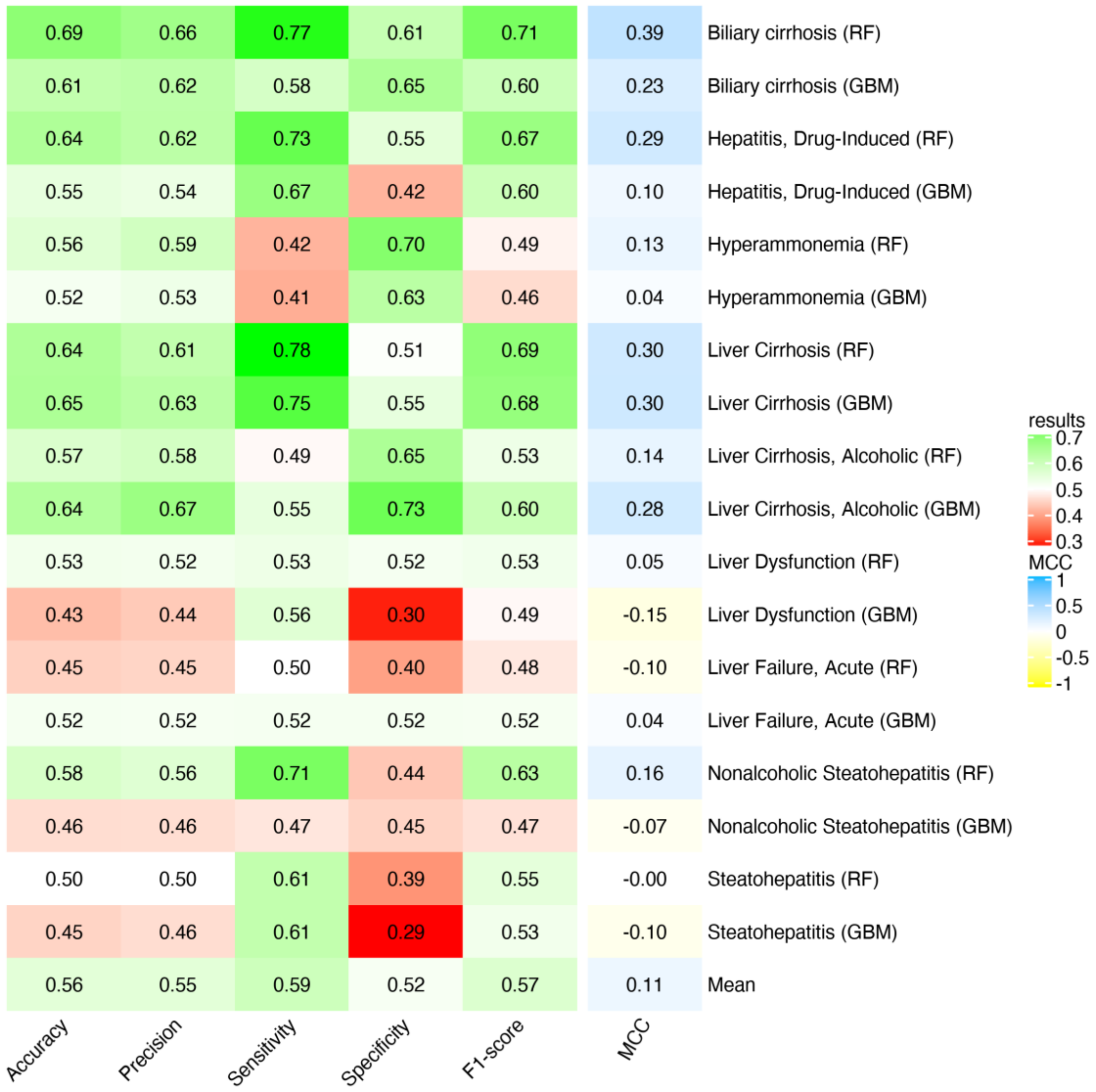

Supplementary Figure 9. Result of the classifiers based on gene sets from DisGeNET DILI phenotypes in the testing set. The machine learning algorithms used are either Random Forest (RF) or Gradient Boosting Machine (GBM). Each row corresponds to the mean performance of 10 models trained using the PHH gene expression of the genes associated to each DILI phenotype. The “Mean” row corresponds to the average performance of each metric for all the phenotypes.

Supplementary Table 1

Supplementary Table 1: List of associations between DILI phenotypes and genes from DisGeNET and GUILDify. The number “1” in the columns DisGeNET or GUILDify indicates that the phenotype-gene association comes from this source, and the number “0” indicates the opposite.

The table is provided as a separated file.

Supplementary Table 2

Supplementary Table 2: List of SMILES from the drugs of the analysis.

The table is provided as a separated file.

Supplementary Table 3

Supplementary Table 3: Tanimoto distance matrix between the drugs of the analysis.

The table is provided as a separated file.

Supplementary Table 4

Supplementary Table 4: List of drug-target associations used in the analysis. The drug-target associations are retrieved from DGIdb, HitPick and SEA. The number “1” indicates a drug-target association, and the number “0” indicates the opposite.

The table is provided as a separated file.

Supplementary Table 5

Supplementary Table 5: Number of the drugs used in each step of the machine learning process. In parenthesis, the number of drugs when using “Targets” feature.

Type of drug	Number of drugs		
	Complete dataset	Training	Testing
DILIrank drugs	175 (172)	72	30
DILI-Concern drugs	124 (121)	36	15
Most-DILI-Concern drugs	37 (36)	11	4
Less-DILI-Concern drugs	87 (85)	25	11
No-DILI-Concern drugs	51 (51)	36	15
Independent hold-out test dataset drugs	55 (53)		

Supplementary Table 6

Supplementary Table 6: List of hepatotoxic genes from the study of *Peng et al. (2019)* and their overlap with the datasets of the article.

The table is provided as a separated file.

Supplementary Table 7

Supplementary Table 7: List of genes from the DILI Landmark gene signature (obtained from a non-parametric Wilcoxon test).

The table is provided as a separated file.

Supplementary Table 8

Supplementary Table 8: List of 20 target proteins that are targeted by the largest number of drugs from the independent hold-out dataset (Ambiguous-DILI drugs). In the table, we provide the number and percentage of interacting drugs from the independent hold-out dataset, and the number and percentage of interacting in total (from the 230 drugs of the dataset).

Target name	Num. drugs interacting (Ambiguous-DILI)	% drugs interacting (Ambiguous-DILI)	Num. drugs interacting (Total)	% drugs interacting (Total)
CYP2D6	18	32.7	92	40.0
CYP3A4	17	30.9	100	43.5
ABCB1	17	30.9	84	36.5
CYP2C9	11	20.0	74	32.2
CYP1A2	11	20.0	59	25.7
DRD2	10	18.2	42	18.3
HTR2A	9	16.4	45	19.6
CYP3A5	8	14.5	46	20.0
ADRA2B	8	14.5	41	17.8
DRD1	8	14.5	34	14.8
ADRA2C	8	14.5	34	14.8
HTR1A	8	14.5	31	13.5
SLC22A1	8	14.5	25	10.9
ESR1	7	12.7	22	9.6
CA1	7	12.7	15	6.5
HTR2C	7	12.7	40	17.4
ADRA2A	7	12.7	37	16.1
HRH1	7	12.7	30	13.0
ADRB1	7	12.7	24	10.4
CYP2C19	6	10.9	40	17.4
